# Bis(cytosinium) aqua­penta­chlorido­indate(III)

**DOI:** 10.1107/S1600536811004235

**Published:** 2011-02-12

**Authors:** Sofiane Bouacida, Ratiba Belhouas, Boubakeur Fantazi, Chaouki Boudaren, Thierry Roisnel

**Affiliations:** a Unité de Recherche de Chimie de l’Environnement et Moléculaire Structurale, CHEMS, Université Mentouri-Constantine, 25000 Algeria; b Centre de Difractométrie X, UMR 6226 CNRS Unité Sciences Chimiques de Rennes, Université de Rennes I, 263 Avenue du général Leclerc, 35042 Rennes, France.

## Abstract

The asymmetric unit of the title compound, (C_4_H_6_N_3_O)_2_[InCl_5_(H_2_O)], comprises two independent cytosinium cations and an aquapentachloridoindate anion. The In^III^ ion is in a slightly distorted octa­hedral coordination geometry. In the crystal, alternating layers of cations and anions are arranged along [010] and are linked *via* inter­molecular N—H⋯O, O—H⋯Cl and N—H⋯Cl hydrogen bonds, forming sheets parallel to (001). Additional stabilization within these sheeets is provided by weak inter­molecular C—H⋯O inter­actions.

## Related literature

For related structures, see: Bouacida (2008 ▶); Bouacida *et al.* (2005 ▶, 2009 ▶); Casellato *et al.* (1995 ▶); Cherouana *et al.* (2003 ▶). For standard bond lengths see: Allen *et al.* (1987 ▶).
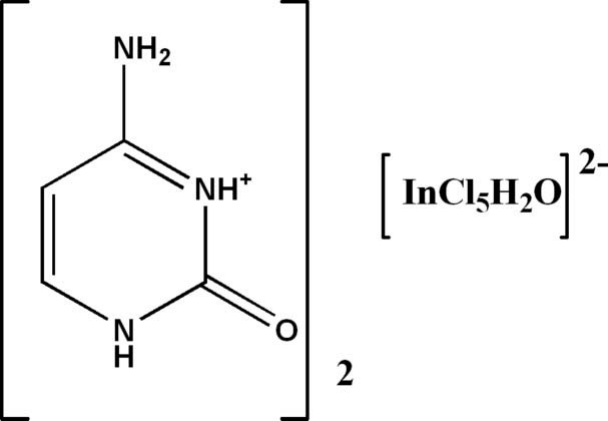


## Experimental

### 

#### Crystal data

(C_4_H_6_N_3_O)_2_[InCl_5_(H_2_O)]
*M* *_r_* = 534.32
Triclinic, 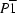

*a* = 6.863 (1) Å
*b* = 10.487 (2) Å
*c* = 12.765 (2) Å
α = 104.608 (1)°
β = 97.998 (1)°
γ = 98.121 (1)°
*V* = 865.3 (2) Å^3^
*Z* = 2
Mo *K*α radiation
μ = 2.16 mm^−1^
*T* = 295 K
0.18 × 0.09 × 0.07 mm

#### Data collection

Nonius KappaCCD diffractometer
18109 measured reflections
3933 independent reflections
3572 reflections with *I* > 2σ(*I*)
*R*_int_ = 0.032

#### Refinement

*R*[*F* ^2^ > 2σ(*F* ^2^)] = 0.020
*wR*(*F* ^2^) = 0.048
*S* = 1.07
3929 reflections
214 parameters
H atoms treated by a mixture of independent and constrained refinement
Δρ_max_ = 0.35 e Å^−3^
Δρ_min_ = −0.61 e Å^−3^

### 

Data collection: *COLLECT* (Nonius, 1998 ▶); cell refinement: *SCALEPACK* (Otwinowski & Minor, 1997 ▶); data reduction: *DENZO* (Otwinowski & Minor, 1997 ▶) and *SCALEPACK*; program(s) used to solve structure: *SIR2002* (Burla *et al.*, 2003 ▶); program(s) used to refine structure: *SHELXL97* (Sheldrick, 2008 ▶); molecular graphics: *PLATON* (Spek, 2009 ▶) and *DIAMOND* (Brandenburg *et al.*, 2001 ▶); software used to prepare material for publication: *WinGX* (Farrugia, 1999 ▶).

## Supplementary Material

Crystal structure: contains datablocks global, I. DOI: 10.1107/S1600536811004235/lh5204sup1.cif

Structure factors: contains datablocks I. DOI: 10.1107/S1600536811004235/lh5204Isup2.hkl

Additional supplementary materials:  crystallographic information; 3D view; checkCIF report

## Figures and Tables

**Table 1:** Hydrogen-bond geometry (Å, °)

*D*—H⋯*A*	*D*—H	H⋯*A*	*D*⋯*A*	*D*—H⋯*A*
O1*W*—H1*W*⋯Cl1^i^	0.80 (3)	2.52 (3)	3.3033 (17)	167 (2)
N2*A*—H2*A*⋯Cl4^ii^	0.86	2.41	3.2185 (18)	156
N2*B*—H2*B*⋯Cl2^iii^	0.86	2.47	3.2774 (18)	157
O1*W*—H2*W*⋯Cl2^ii^	0.78 (3)	2.49 (3)	3.2667 (18)	174 (3)
N6*A*—H6*A*⋯Cl3^iii^	0.86	2.37	3.2104 (17)	164
N6*B*—H6*B*⋯Cl5^ii^	0.86	2.38	3.2160 (18)	163
N7*A*—H71*A*⋯O1*A*^i^	0.86	2.19	2.965 (3)	150
N7*B*—H71*B*⋯O1*W*^iii^	0.86	2.38	3.226 (3)	168
N7*A*—H72*A*⋯Cl1^ii^	0.86	2.69	3.471 (2)	152
N7*B*—H72*B*⋯O1*B*^iv^	0.86	2.22	2.987 (3)	149
C4*A*—H4*A*⋯O1*A*^i^	0.93	2.30	3.068 (3)	140
C4*B*—H4*B*⋯O1*B*^iv^	0.93	2.28	3.051 (3)	140

Symmetry codes: (i) 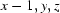; (ii) 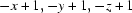; (iii) 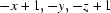; (iv) 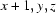.

**Fig. 1.**
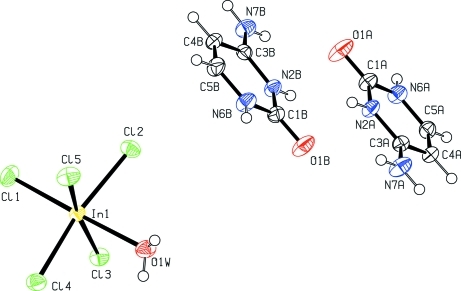
The asymmetric unit of the title compound with displacement ellipsoids drawn at the 50% probability level.

**Fig. 2.**
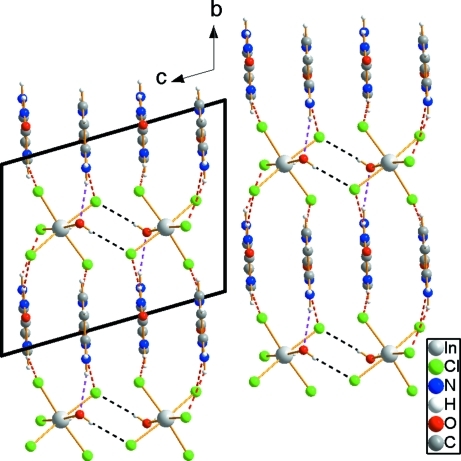
Part of the crystal structure with hydrogen bonds shown as dashed lines.

## supplementary crystallographic information

### Comment

The title compound, was prepared as part of our ongoing studies of hydrogen-bonding interaction in the crystal structures of protonated amines (Bouacida, 2008; Bouacida *et al.*, 2009). The asymmetric unit of the title compound (I) is shown in Fig. 1.  The bond distances (Allen *et al.* 1987) and angles are within the ranges of accepted values. In the title compound the imine N atom is protonated as in other related structures (Bouacida *et al.*, 2005; Casellato, *et al.* 1995; Cherouana *et al.*, 2003). The In atom is six-coordinated (by five chlorine atoms and one water molecule) forming a slightly-distorted octahedral geometry. In the crystal structure alternating layers of cations and anions are arranged along [010] and are linked *via* intermolecular N—H···O, O—H···Cl and N—H···Cl hydrogen bonds to form a two-dimensional sheets parallel to (001) (see Fig. 2). Additional stabilization within these sheeets is provided by weak intermolecular C—H···O interactions.

### Experimental

A solution of 1 mmol InCl_3_ and 2 mmol cytosine in hydrochloric acid was slowly evaporated to dryness over a period of two weeks yielding red crystals suitable for X-ray diffraction.

### Refinement

All H atoms were visible in differnce Fourier maps but were introduced in calculated positions and treated as riding on C and N atoms with C—H = 0.93Å and N—H = 0.86Å and *U*_iso_(H) = 1.2(C,N). The water H atoms were located in a difference Fourier map and their positions were refined with *U*_iso_(H) =1.5 *U*_eq_(O).

### Crystal data

**Table:**

(C_4_H_6_N_3_O)_2_[InCl_5_(H2O)]	*Z* = 2
*M**_r_* = 534.32	*F*(000) = 524
Triclinic, *P*1	*D*_x_ = 2.051 Mg m^−^^3^
*a* = 6.863 (1) Å	Mo *K*α radiation, λ = 0.71073 Å
*b* = 10.487 (2) Å	Cell parameters from 8762 reflections
*c* = 12.765 (2) Å	θ = 3.1–27.5°
α = 104.608 (1)°	µ = 2.16 mm^−^^1^
β = 97.998 (1)°	*T* = 295 K
γ = 98.121 (1)°	Needle, red
*V* = 865.3 (2) Å^3^	0.18 × 0.09 × 0.07 mm

### Data collection

**Table:**

Nonius KappaCCD diffractometer	*R*_int_ = 0.032
graphite	θ_max_ = 27.6°, θ_min_ = 1.7°
CCD rotation images, thick slices scans	*h* = −8→8
18109 measured reflections	*k* = −13→13
3933 independent reflections	*l* = −16→16
3572 reflections with *I* > 2σ(*I*)	

### Refinement

**Table:**

Refinement on *F*^2^	Primary atom site location: structure-invariant direct methods
Least-squares matrix: full	Secondary atom site location: difference Fourier map
*R*[*F*^2^ > 2σ(*F*^2^)] = 0.02	Hydrogen site location: inferred from neighbouring sites
*wR*(*F*^2^) = 0.048	H atoms treated by a mixture of independent and constrained refinement
*S* = 1.07	*w* = 1/[σ^2^(*F*_o_^2^) + (0.0229*P*)^2^ + 0.0464*P*] where *P* = (*F*_o_^2^ + 2*F*_c_^2^)/3
3929 reflections	(Δ/σ)_max_ = 0.002
214 parameters	Δρ_max_ = 0.35 e Å^−^^3^
0 restraints	Δρ_min_ = −0.61 e Å^−^^3^

### Special details

**Table:**

Geometry. All e.s.d.'s (except the e.s.d. in the dihedral angle between two l.s. planes) are estimated using the full covariance matrix. The cell e.s.d.'s are taken into account individually in the estimation of e.s.d.'s in distances, angles and torsion angles; correlations between e.s.d.'s in cell parameters are only used when they are defined by crystal symmetry. An approximate (isotropic) treatment of cell e.s.d.'s is used for estimating e.s.d.'s involving l.s. planes.
Refinement. Refinement of *F*^2^ against ALL reflections. The weighted *R*-factor *wR* and goodness of fit *S* are based on *F*^2^, conventional *R*-factors *R* are based on *F*, with *F* set to zero for negative *F*^2^. The threshold expression of *F*^2^ > σ(*F*^2^) is used only for calculating *R*-factors(gt) *etc*. and is not relevant to the choice of reflections for refinement. *R*-factors based on *F*^2^ are statistically about twice as large as those based on *F*, and *R*- factors based on ALL data will be even larger.4 bad reflections were omitted from the refinement

### Fractional atomic coordinates and isotropic or equivalent isotropic displacement parameters (Å^2^)

**Table:**

	*x*	*y*	*z*	*U*_iso_*/*U*_eq_	
In1	0.550968 (18)	0.423292 (12)	0.265050 (10)	0.02044 (5)	
Cl2	0.68990 (8)	0.33651 (5)	0.42094 (4)	0.03022 (11)	
Cl3	0.35973 (7)	0.19643 (4)	0.16412 (4)	0.02749 (11)	
Cl4	0.36901 (7)	0.51814 (5)	0.13047 (4)	0.03375 (12)	
Cl5	0.68860 (8)	0.65285 (5)	0.38414 (5)	0.03400 (12)	
Cl1	0.84669 (8)	0.39953 (6)	0.17617 (5)	0.03882 (13)	
N2B	0.6503 (2)	−0.06652 (16)	0.62369 (13)	0.0249 (4)	
H2B	0.5921	−0.1469	0.6175	0.03*	
O1B	0.3547 (2)	0.00633 (15)	0.63256 (14)	0.0405 (4)	
N6A	0.3443 (2)	0.01389 (16)	0.88504 (14)	0.0282 (4)	
H6A	0.411	−0.0502	0.8804	0.034*	
O1A	0.6221 (2)	0.16293 (15)	0.89141 (15)	0.0455 (4)	
N6B	0.6325 (2)	0.15524 (17)	0.63714 (15)	0.0316 (4)	
H6B	0.5662	0.2195	0.641	0.038*	
N7B	0.9460 (2)	−0.14472 (16)	0.62174 (14)	0.0304 (4)	
H71B	0.8827	−0.2227	0.619	0.037*	
H72B	1.0728	−0.1321	0.6225	0.037*	
C4B	0.9437 (3)	0.08585 (19)	0.63066 (16)	0.0264 (4)	
H4B	1.08	0.1051	0.6303	0.032*	
C3B	0.8491 (3)	−0.04516 (19)	0.62486 (15)	0.0227 (4)	
C4A	0.0348 (3)	0.08195 (19)	0.89372 (16)	0.0258 (4)	
H4A	−0.1012	0.0624	0.8951	0.031*	
C5B	0.8318 (3)	0.1819 (2)	0.63676 (17)	0.0302 (5)	
H5B	0.8922	0.2685	0.6408	0.036*	
C1B	0.5332 (3)	0.0312 (2)	0.63174 (16)	0.0266 (4)	
C3A	0.1280 (3)	0.21374 (18)	0.90100 (15)	0.0234 (4)	
O1W	0.2642 (2)	0.42641 (16)	0.35025 (13)	0.0319 (3)	
H1W	0.166 (4)	0.433 (2)	0.312 (2)	0.048*	
H2W	0.272 (4)	0.487 (3)	0.402 (2)	0.048*	
C5A	0.1454 (3)	−0.01407 (19)	0.88486 (16)	0.0267 (4)	
H3A	0.0849	−0.1015	0.8785	0.032*	
C1A	0.4435 (3)	0.1381 (2)	0.89219 (17)	0.0284 (4)	
N7A	0.0296 (3)	0.31296 (17)	0.90638 (15)	0.0354 (4)	
H72A	0.091	0.3911	0.9082	0.042*	
H71A	−0.0963	0.2999	0.9081	0.042*	
N2A	0.3263 (2)	0.23531 (16)	0.89832 (14)	0.0258 (4)	
H2A	0.3828	0.3148	0.9006	0.031*	

### Atomic displacement parameters (Å^2^)

**Table:**

	*U*^11^	*U*^22^	*U*^33^	*U*^12^	*U*^13^	*U*^23^
In1	0.01960 (8)	0.01626 (7)	0.02516 (8)	0.00200 (5)	0.00384 (5)	0.00607 (5)
Cl2	0.0331 (3)	0.0272 (2)	0.0304 (3)	0.0062 (2)	0.0002 (2)	0.0106 (2)
Cl3	0.0290 (3)	0.0184 (2)	0.0310 (3)	0.00087 (18)	0.0023 (2)	0.00312 (19)
Cl4	0.0287 (3)	0.0312 (3)	0.0428 (3)	0.0015 (2)	−0.0020 (2)	0.0198 (2)
Cl5	0.0313 (3)	0.0185 (2)	0.0444 (3)	−0.0001 (2)	−0.0013 (2)	0.0017 (2)
Cl1	0.0277 (3)	0.0490 (3)	0.0454 (3)	0.0106 (2)	0.0166 (2)	0.0159 (3)
N2B	0.0211 (8)	0.0216 (8)	0.0346 (9)	0.0037 (6)	0.0062 (7)	0.0118 (7)
O1B	0.0207 (8)	0.0379 (9)	0.0675 (11)	0.0080 (7)	0.0129 (7)	0.0187 (8)
N6A	0.0253 (9)	0.0235 (8)	0.0384 (10)	0.0089 (7)	0.0066 (8)	0.0101 (7)
O1A	0.0206 (8)	0.0371 (9)	0.0813 (13)	0.0066 (7)	0.0145 (8)	0.0175 (9)
N6B	0.0244 (9)	0.0256 (9)	0.0501 (11)	0.0096 (7)	0.0088 (8)	0.0162 (8)
N7B	0.0255 (9)	0.0268 (9)	0.0386 (10)	0.0071 (7)	0.0055 (8)	0.0072 (8)
C4B	0.0183 (9)	0.0304 (11)	0.0312 (11)	0.0022 (8)	0.0048 (8)	0.0113 (9)
C3B	0.0229 (10)	0.0257 (10)	0.0193 (9)	0.0051 (8)	0.0034 (7)	0.0060 (8)
C4A	0.0180 (9)	0.0301 (11)	0.0288 (11)	0.0011 (8)	0.0043 (8)	0.0093 (8)
C5B	0.0268 (11)	0.0241 (10)	0.0402 (12)	−0.0012 (8)	0.0051 (9)	0.0135 (9)
C1B	0.0215 (10)	0.0310 (11)	0.0306 (11)	0.0079 (8)	0.0062 (8)	0.0122 (9)
C3A	0.0212 (9)	0.0230 (9)	0.0243 (10)	0.0035 (8)	0.0038 (8)	0.0041 (8)
O1W	0.0262 (8)	0.0365 (9)	0.0297 (8)	0.0082 (7)	0.0045 (6)	0.0022 (6)
C5A	0.0263 (10)	0.0246 (10)	0.0276 (11)	−0.0005 (8)	0.0041 (8)	0.0079 (8)
C1A	0.0219 (10)	0.0284 (10)	0.0353 (11)	0.0061 (8)	0.0069 (9)	0.0078 (9)
N7A	0.0239 (9)	0.0265 (9)	0.0529 (12)	0.0052 (7)	0.0076 (8)	0.0054 (8)
N2A	0.0191 (8)	0.0216 (8)	0.0372 (10)	0.0015 (6)	0.0062 (7)	0.0095 (7)

### Geometric parameters (Å, °)

**Table:**

In1—O1W	2.3776 (15)	N7B—H71B	0.86
In1—Cl1	2.4718 (6)	N7B—H72B	0.86
In1—Cl5	2.4720 (6)	C4B—C5B	1.344 (3)
In1—Cl4	2.4730 (6)	C4B—C3B	1.413 (3)
In1—Cl3	2.4787 (6)	C4B—H4B	0.93
In1—Cl2	2.5155 (6)	C4A—C5A	1.337 (3)
N2B—C3B	1.349 (2)	C4A—C3A	1.413 (3)
N2B—C1B	1.381 (2)	C4A—H4A	0.93
N2B—H2B	0.86	C5B—H5B	0.93
O1B—C1B	1.218 (2)	C3A—N7A	1.311 (2)
N6A—C5A	1.354 (2)	C3A—N2A	1.355 (2)
N6A—C1A	1.356 (3)	O1W—H1W	0.80 (2)
N6A—H6A	0.86	O1W—H2W	0.78 (3)
O1A—C1A	1.218 (2)	C5A—H3A	0.93
N6B—C5B	1.357 (2)	C1A—N2A	1.379 (2)
N6B—C1B	1.361 (2)	N7A—H72A	0.86
N6B—H6B	0.86	N7A—H71A	0.86
N7B—C3B	1.310 (2)	N2A—H2A	0.86
			
O1W—In1—Cl1	175.07 (4)	N7B—C3B—N2B	119.31 (17)
O1W—In1—Cl5	88.65 (4)	N7B—C3B—C4B	123.05 (17)
Cl1—In1—Cl5	95.26 (2)	N2B—C3B—C4B	117.64 (17)
O1W—In1—Cl4	86.45 (4)	C5A—C4A—C3A	118.82 (17)
Cl1—In1—Cl4	96.55 (2)	C5A—C4A—H4A	120.6
Cl5—In1—Cl4	89.56 (2)	C3A—C4A—H4A	120.6
O1W—In1—Cl3	80.65 (4)	C4B—C5B—N6B	121.53 (18)
Cl1—In1—Cl3	95.42 (2)	C4B—C5B—H5B	119.2
Cl5—In1—Cl3	169.296 (17)	N6B—C5B—H5B	119.2
Cl4—In1—Cl3	89.95 (2)	O1B—C1B—N6B	123.31 (18)
O1W—In1—Cl2	83.91 (4)	O1B—C1B—N2B	121.90 (18)
Cl1—In1—Cl2	93.21 (2)	N6B—C1B—N2B	114.78 (16)
Cl5—In1—Cl2	88.07 (2)	N7A—C3A—N2A	119.53 (17)
Cl4—In1—Cl2	170.125 (18)	N7A—C3A—C4A	122.86 (18)
Cl3—In1—Cl2	90.60 (2)	N2A—C3A—C4A	117.57 (17)
C3B—N2B—C1B	124.87 (16)	In1—O1W—H1W	114.4 (18)
C3B—N2B—H2B	117.6	In1—O1W—H2W	115.7 (19)
C1B—N2B—H2B	117.6	H1W—O1W—H2W	101 (2)
C5A—N6A—C1A	123.24 (17)	C4A—C5A—N6A	121.06 (18)
C5A—N6A—H6A	118.4	C4A—C5A—H3A	119.5
C1A—N6A—H6A	118.4	N6A—C5A—H3A	119.5
C5B—N6B—C1B	122.77 (17)	O1A—C1A—N6A	123.22 (18)
C5B—N6B—H6B	118.6	O1A—C1A—N2A	121.78 (18)
C1B—N6B—H6B	118.6	N6A—C1A—N2A	114.99 (17)
C3B—N7B—H71B	120	C3A—N7A—H72A	120
C3B—N7B—H72B	120	C3A—N7A—H71A	120
H71B—N7B—H72B	120	H72A—N7A—H71A	120
C5B—C4B—C3B	118.37 (18)	C3A—N2A—C1A	124.27 (16)
C5B—C4B—H4B	120.8	C3A—N2A—H2A	117.9
C3B—C4B—H4B	120.8	C1A—N2A—H2A	117.9
			
C1B—N2B—C3B—N7B	176.87 (18)	C5A—C4A—C3A—N7A	177.79 (19)
C1B—N2B—C3B—C4B	−2.5 (3)	C5A—C4A—C3A—N2A	0.1 (3)
C5B—C4B—C3B—N7B	−178.07 (19)	C3A—C4A—C5A—N6A	1.3 (3)
C5B—C4B—C3B—N2B	1.2 (3)	C1A—N6A—C5A—C4A	−1.1 (3)
C3B—C4B—C5B—N6B	−0.2 (3)	C5A—N6A—C1A—O1A	−179.4 (2)
C1B—N6B—C5B—C4B	0.2 (3)	C5A—N6A—C1A—N2A	−0.6 (3)
C5B—N6B—C1B—O1B	179.6 (2)	N7A—C3A—N2A—C1A	−179.71 (19)
C5B—N6B—C1B—N2B	−1.2 (3)	C4A—C3A—N2A—C1A	−2.0 (3)
C3B—N2B—C1B—O1B	−178.40 (19)	O1A—C1A—N2A—C3A	−179.1 (2)
C3B—N2B—C1B—N6B	2.4 (3)	N6A—C1A—N2A—C3A	2.2 (3)

### Hydrogen-bond geometry (Å, °)

**Table:**

*D*—H···*A*	*D*—H	H···*A*	*D*···*A*	*D*—H···*A*
O1W—H1W···Cl1^i^	0.80 (3)	2.52 (3)	3.3033 (17)	167 (2)
N2A—H2A···Cl4^ii^	0.86	2.41	3.2185 (18)	156
N2B—H2B···Cl2^iii^	0.86	2.47	3.2774 (18)	157
O1W—H2W···Cl2^ii^	0.78 (3)	2.49 (3)	3.2667 (18)	174 (3)
N6A—H6A···Cl3^iii^	0.86	2.37	3.2104 (17)	164
N6B—H6B···Cl5^ii^	0.86	2.38	3.2160 (18)	163
N7A—H71A···O1A^i^	0.86	2.19	2.965 (3)	150
N7B—H71B···O1W^iii^	0.86	2.38	3.226 (3)	168
N7A—H72A···Cl1^ii^	0.86	2.69	3.471 (2)	152
N7B—H72B···O1B^iv^	0.86	2.22	2.987 (3)	149
C4A—H4A···O1A^i^	0.93	2.30	3.068 (3)	140
C4B—H4B···O1B^iv^	0.93	2.28	3.051 (3)	140

Symmetry codes: (i) *x*−1, *y*, *z*; (ii) −*x*+1, −*y*+1, −*z*+1; (iii) −*x*+1, −*y*, −*z*+1; (iv) *x*+1, *y*, *z*.
